# Supplementary figures and images for: Evaluating the adaptive evolutionary convergence of carnivorous plant taxa through functional genomics
Source: PeerJ. 2018 Jan 31;6:e4322. doi: 10.7717/peerj.4322 (PMC5797450; doi:10.7717/peerj.4322)

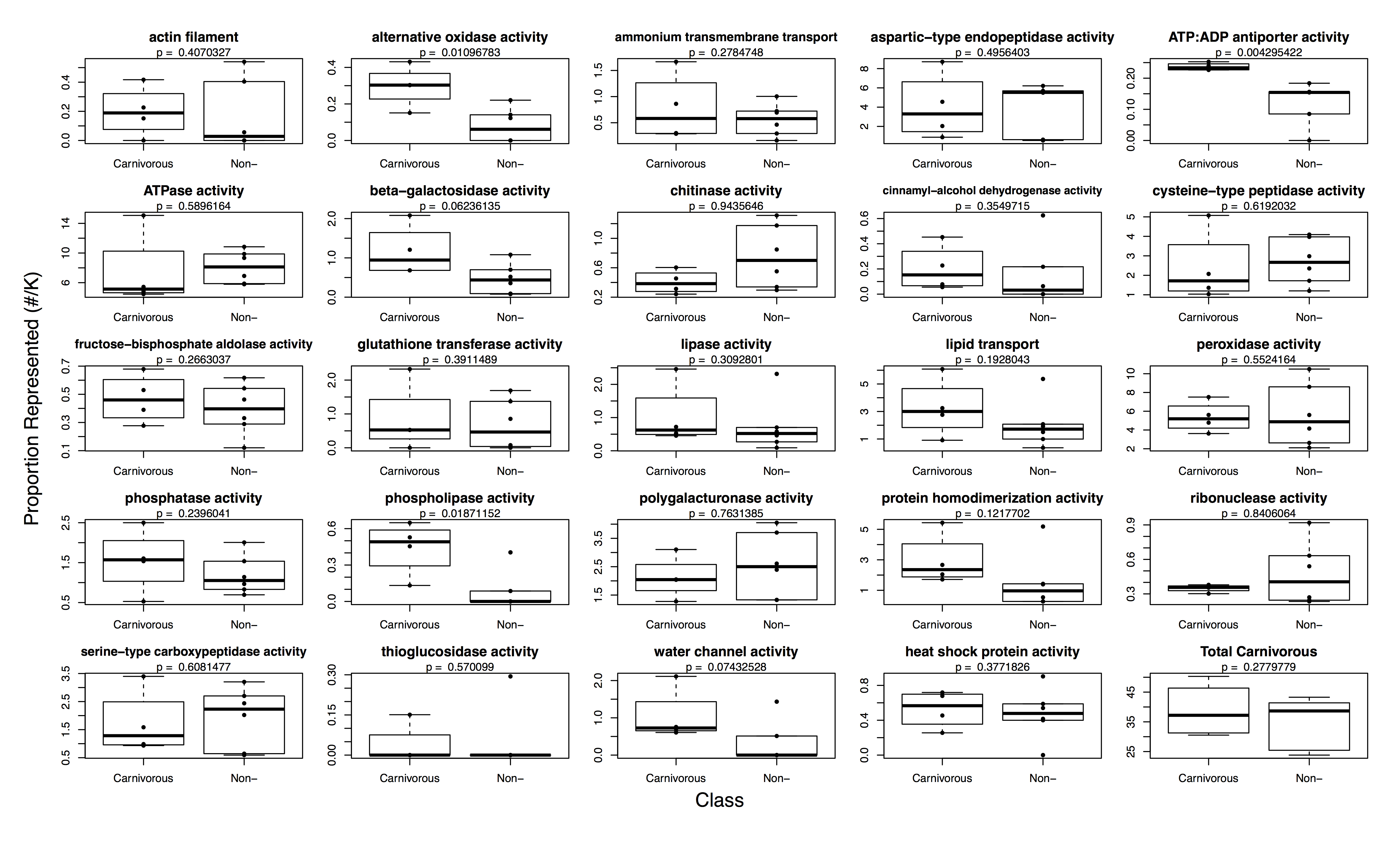

Supplement: Figure S1 — Each boxplot depicts one of 24 comparisons between the relative proportion of a carnivory-associated function in carnivorous vs. non-carnivorous plants, plus the sum of all these functions. Dots show the position and effects of individual samples within each distribution. [file peerj-06-4322-s001.png]

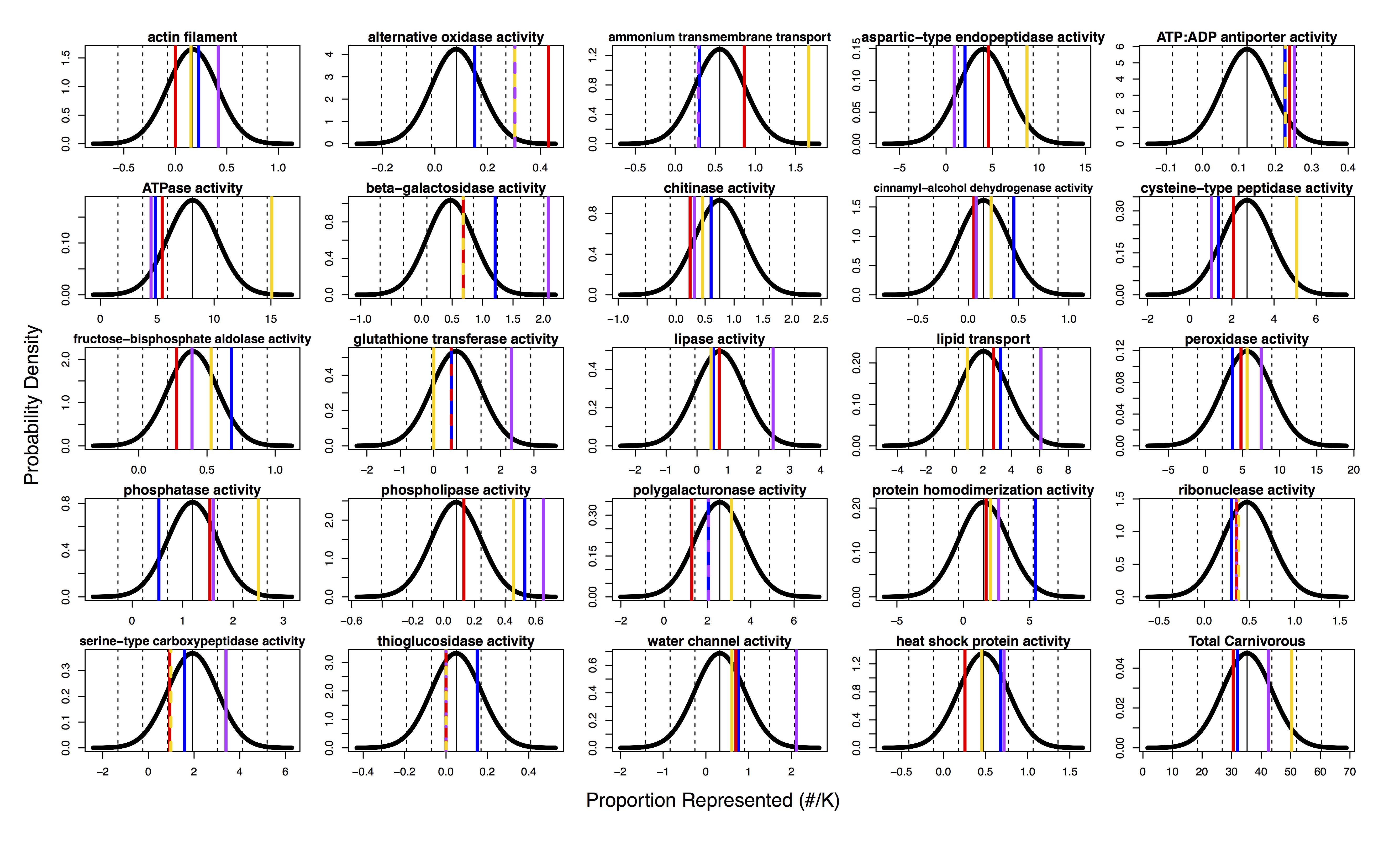

Supplement: Figure S2 — Each normal distribution represents the range of values found in non-carnivorous taxa for a function analyzed. Colored lines each indicate the value found for a carnivorous taxon (Blue: Genlisea aurea; Red: Drosera capensis; Yellow: Utricularia gibba; Purple: Cephalotus follicularis). Black vertical lines indicate mean (solid) and standard deviations from the mean (dashed). [file peerj-06-4322-s002.png]
