# Supplementary material for: Evaluating the adaptive evolutionary convergence of carnivorous plant taxa through functional genomics
Source: PeerJ. 2018 Jan 31;6:e4322. doi: 10.7717/peerj.4322 (PMC5797450; doi:10.7717/peerj.4322)
Supplement: Table S1 — Where zones of the plant are given (e.g., trap, fluid, or secretion), analysis was localized to that specific zone or accounted for differential expression. Where only the taxon is specified, analysis considered the whole plant. Information from: Rottloff et al. (2016) [1], Schulze et al. (2012) [2], Scherzer et al. (2013) [3], Owen (1999) [4], Böhm et al. (2016) [5], Ibarra-Laclette et al. (2011) [6], and An, Fukusaki & Kobayashi (2002) [7]. [file peerj-06-4322-s003.docx]

| **Function** | **Identified In** | **Term Assigned** | **Function** | **Identified In** | **Term Assigned** |
| --- | --- | --- | --- | --- | --- |
| Acid Chitinase | *Nepenthes* pitcher fluid [1] | *chitinase activity* | Heat Shock Protein | *Dionaea* *muscipula* secretion [2] | heat shock protein activity |
| Actin | *Dionaea* *muscipula* secretion [2] | *actin filament* | Histone Protein | *Dionaea* *muscipula* secretion [2] | No Match |
| ADP/ATP Carrier | *Dionaea* *muscipula* secretion [2] | *ATP:ADP antiporter activity* | HKT1 Sodium Channel | *Dionaea* *muscipula* traps [5] | *sodium ion transmembrane transporter activity* |
| alpha-Galactosidase | *Nepenthes* pitcher fluid [1] | *alpha-galactosidase activity* | Lipase | *Dionaea* *muscipula* secretion [2]; *Nepenthes* pitcher fluid [1] | *lipase activity* |
| Alternative Oxidase 1A | *Utricularia* *gibba* trap [6] | *alternative oxidase activity* | Lipid Transfer Protein | *Dionaea* *muscipula* secretion [2]; *Utricularia* *gibba* trap [6]; *Nepenthes* pitcher fluid [1] | *lipid transport* |
| AMT1 | *Dionaea* *muscipula* [3] | *ammonium transmembrane transport* | Methylammonium Transmembrane Channel | *Utricularia* gibba shoot [6] | *methylammonium channel activity* |
| Aspartyl Protease | *Dionaea* *muscipula* secretion [2]; *Nepenthes* pitcher fluid [1, 7] | *aspartic-type endopeptidase activity* | Nucleotide phosphodiesterase | *Dionaea* *muscipula* secretion [2]; *Nepenthes* pitcher fluid [1] | *cyclic-nucleotide phosphodiesterase activity* |
| ATP Synthase | *Dionaea* *muscipula* secretion [2] | *ATPase activity* | Osmotin-like Protein | *Dionaea* *muscipula* secretion [2] | *water channel activity* |
| ATPase | *Dionaea* *muscipula* secretion [2] | *ATPase activity* | Pathogenesis-related Protein | *Dionaea* *muscipula* secretion [2] | No Match |
| beta-1,3-Glucanase | *Nepenthes* pitcher fluid [1] | *beta-glucanase activity* | Peroxidase | *Dionaea* *muscipula* secretion [2]; *Utricularia* *gibba* shoot [6]; *Nepenthes* pitcher fluid [1] | *peroxidase activity* |
| beta-Galactosidase | *Nepenthes* pitcher fluid [1] | *beta-galactosidase activity* | Phosphatase | *Dionaea* *muscipula* secretion [2] | *phosphatase activity* |
| Cationic peroxidase | *Nepenthes* pitcher fluid [1] | *peroxidase activity* | Phospholipase | *Dionaea* *muscipula* secretion [2] | *phospholipase activity* |
| Chitinase | *Dionaea* *muscipula* secretion [2]; *Nepenthes* pitcher fluid [1] | *chitinase activity* | Plasma membrane water channel | *Utricularia* *gibba* shoot [6] | *water channel activity* |
| Cinnamyl Alcohol Dehydrogenase | *Dionaea* *muscipula* secretion [2] | *cinnamyl-alcohol dehydrogenase activity* | Polygalacturonase | *Utricularia* *gibba* shoot [6] | *polygalacturonase activity* |
| Cysteine Protease | *Dionaea* *muscipula* secretion [2]; *Utricularia* *gibba* trap [6] | *cysteine-type peptidase activity* | Polygalacturonase Inhibitor | *Dionaea* *muscipula* secretion [2] | *polygalacturonase inhibitor activity* |
| Elongation Factor | *Dionaea* *muscipula* secretion [2] | No Match | Protein homodimerization | *Utricularia* *gibba* shoot [6] | *protein homodimerization activity* |
| Embryogenesis Protein | *Dionaea* *muscipula* secretion [2] | No Match | Protein phosphatase | *Nepenthes* pitcher fluid [1] | *phosphatase activity* |
| Endonuclease | *Dionaea* *muscipula* secretion [2] | *endonuclease complex* | Protodermal Factor | *Dionaea* *muscipula* secretion [2] | No Match |
| Formate Dehydrogenase | *Dionaea* *muscipula* secretion [2] | *formate dehydrogenase complex* | Ribonuclease | *Dionaea* *muscipula* secretion [2]; *Utricularia* *gibba* trap [6] | *ribonuclease activity* |
| Fructose-bisphosphate Aldolase | *Dionaea* *muscipula* secretion [2] | *fructose-bisphosphate aldolase activity* | Serine Carboxypeptidase | *Dionaea* *muscipula* secretion [2]; *Utricularia* *gibba* trap [6]; *Nepenthes* pitcher fluid [1] | *serine-type carboxypeptidase activity* |
| G3P Dehydrogenase | *Dionaea* *muscipula* secretion [2] | No Match | Stigma-specific Protein | *Dionaea* *muscipula* secretion [2] | No Match |
| G-Factor Binding Protein | *Dionaea* muscipula secretion [2] | No Match | Superoxide Dismutase | *Dionaea* *muscipula* secretion [2] | *superoxide dismutase activity* |
| Glucanase | *Dionaea* muscipula secretion [2]; *Nepenthes* pitcher fluid [1] | No Match | Symplast | *Nepenthes* pitcher glands [4] | *symplast* |
| Glucosidase | *Nepenthes* pitcher fluid [1] | *glucosidase complex* | Thioglucosidase | *Dionaea* *muscipula* secretion [2] | *thioglucosidase activity* |
| Glutathione Transferase | *Dionaea* *muscipula* secretion [2] | *glutathione transferase activity* | Thiol Protease | *Utricularia* gibba trap [6] | No Match |
| GPI-Anchored Protein Precursor | *Dionaea* *muscipula* secretion [2] | No Match | Ubiquitin Extension Protein | *Dionaea* *muscipula* secretion [2] | No Match |
| G-Protein Suppressor | *Dionaea* *muscipula* secretion [2] |  | Xylosidase | *Nepenthes* pitcher fluid [1] | *xylanase activity* |
