# Supplementary material for: Evaluating the adaptive evolutionary convergence of carnivorous plant taxa through functional genomics
Source: PeerJ. 2018 Jan 31;6:e4322. doi: 10.7717/peerj.4322 (PMC5797450; doi:10.7717/peerj.4322)
Supplement: Table S2 — The “Adjustment” column indicates the value that BLAST proportions are multiplied by to match the value distribution expected of their GenBank counterparts. Nullset symbols indicate functions for which one or both methods detected zero instances, preventing adjustment calculations. Hyphens indicate values that are calculated individually for each sample, using the sum of other post-adjustment values. [file peerj-06-4322-s004.docx]

| **Function** | **GenBank #** | **GenBank %*10** | **BLAST #** | **BLAST %*10** | **Adjustment** |
| --- | --- | --- | --- | --- | --- |
| *actin filament* | 11 | 0.40 | 4 | 0.19 | 2.15 |
| *alpha-galactosidase activity* | 4 | 0.15 | 0 | 0 | *∅* |
| *alternative oxidase activity* | 6 | 0.22 | 4 | 0.19 | 1.17 |
| *ammonium transmembrane transport* | 8 | 0.29 | 7 | 0.33 | 0.89 |
| *aspartic-type endopeptidase activity* | 17 | 0.62 | 93 | 4.37 | 0.14 |
| *ATP:ADP antiporter activity* | 5 | 0.18 | 6 | 0.28 | 0.65 |
| *ATPase activity* | 188 | 6.91 | 369 | 17.34 | 0.40 |
| *beta-galactosidase activity* | 19 | 0.70 | 12 | 0.56 | 1.24 |
| *beta-glucanase activity* | 0 | 0 | 0 | 0 | *∅* |
| *chitinase activity* | 15 | 0.55 | 29 | 1.36 | 0.40 |
| *cinnamyl-alcohol dehydrogenase activity* | 17 | 0.62 | 11 | 0.52 | 1.21 |
| *cyclic-nucleotide phosphodiesterase activity* | 1 | 0.04 | 0 | 0 | *∅* |
| *cysteine-type peptidase activity* | 64 | 2.35 | 122 | 5.73 | 0.41 |
| *endonuclease complex* | 0 | 0 | 0 | 0 | *∅* |
| *formate dehydrogenase complex* | 0 | 0 | 0 | 0 | *∅* |
| *fructose-bisphosphate aldolase activity* | 9 | 0.33 | 7 | 0.33 | 1.01 |
| *glucosidase complex* | 0 | 0 | 0 | 0 | *∅* |
| *glutathione transferase activity* | 46 | 1.69 | 22 | 1.03 | 1.64 |
| *lipase activity* | 63 | 2.32 | 22 | 1.03 | 2.24 |
| *lipid transport* | 146 | 5.37 | 57 | 2.68 | 2.00 |
| *methylammonium channel activity* | 0 | 0 | 0 | 0 | *∅* |
| *peroxidase activity* | 113 | 4.15 | 73 | 3.43 | 1.21 |
| *phosphatase activity* | 31 | 1.14 | 42 | 1.97 | 0.58 |
| *phospholipase activity* | 11 | 0.40 | 6 | 0.28 | 1.43 |
| *polygalacturonase activity* | 71 | 2.61 | 70 | 3.29 | 0.79 |
| *polygalacturonase inhibitor activity* | 2 | 0.07 | 2 | 0.09 | 0.78 |
| *protein homodimerization activity* | 141 | 5.18 | 139 | 6.53 | 0.79 |
| *ribonuclease activity* | 25 | 0.92 | 25 | 1.17 | 0.78 |
| *serine-type carboxypeptidase activity* | 55 | 2.02 | 36 | 1.69 | 1.19 |
| *sodium ion transmembrane transporter activity* | 0 | 0 | 0 | 0 | *∅* |
| *superoxide dismutase activity* | 5 | 0.18 | 0 | 0 | *∅* |
| *symplast* | 0 | 0 | 0 | 0 | *∅* |
| *thioglucosidase activity* | 8 | 0.29 | 1 | 0.05 | 6.26 |
| *water channel activity* | 39 | 1.43 | 14 | 0.66 | 2.18 |
| *xylanase activity* | 0 | 0 | 0 | 0 | *∅* |
| *heat shock protein activity* | 16 | 0.59 | 9 | 0.42 | 1.39 |
| None of the Above | 26091 | 959.02 | 20126 | 945.86 | *-* |
| Total Carnivorous | 1115 | 40.98 | 1152 | 54.14 | *-* |
| Total | 27206 | - | 21278 | - | 1.28 |
