# Supplementary material for: Evaluating the adaptive evolutionary convergence of carnivorous plant taxa through functional genomics
Source: PeerJ. 2018 Jan 31;6:e4322. doi: 10.7717/peerj.4322 (PMC5797450; doi:10.7717/peerj.4322)
Supplement: Table S3 — Functions are ordered by their representation on average (given in far-right column), from most common to most rare. “Other” indicates the total of the nine rarest carnivory-associated functions, listed with indentation. [Abbreviation key: CFOL –Cephalotus follicularis; DCAP –Drosera capensis; GAUR –Genlisea aurea; UGIB –Utricularia gibba; ACHI –Actinidia chinensis; ATHA –Arabidopsis thaliana; BHYG –Boea hygrometrica; GSOJ –Glycine soja; OSAT –Oryza sativa; OTEN –Ocimum tenuiflorum]. [file peerj-06-4322-s005.docx]

|  | **CFOL** | **DCAP** | **GAUR** | **UGIB** | **ACHI** | **ATHA** | **BHYG** | **GSOJ** | **OSAT** | **OTEN** | **Average** |
| --- | --- | --- | --- | --- | --- | --- | --- | --- | --- | --- | --- |
| ATPase activity | 10.50% | 17.79% | 15.09% | 29.79% | 22.76% | 16.73% | 27.59% | 22.82% | 24.54% | 24.66% | 21.23% |
| peroxidase activity | 17.70% | 15.65% | 11.32% | 11.08% | 10.32% | 10.05% | 14.25% | 19.88% | 27.59% | 8.83% | 14.67% |
| aspartic-type endopeptidase activity | 2.07% | 14.92% | 6.37% | 17.22% | 21.88% | 1.51% | 14.48% | 14.35% | 1.42% | 23.09% | 11.73% |
| lipid transport | 2.44% | 6.79% | 4.25% | 10.03% | 4.72% | 5.69% | 7.59% | 9.18% | 10.75% | 7.22% | 6.87% |
| cysteine-type peptidase activity | 14.34% | 9.04% | 10.14% | 1.80% | 3.91% | 12.99% | 5.29% | 3.48% | 5.07% | 1.51% | 6.76% |
| polygalacturonase activity | 4.83% | 4.17% | 6.37% | 6.14% | 5.22% | 6.32% | 9.43% | 9.36% | 6.29% | 5.59% | 6.37% |
| protein homodimerization activity | 6.28% | 5.61% | 16.98% | 4.04% | 5.46% | 12.54% | 0.69% | 0.62% | 1.42% | 5.99% | 5.96% |
| serine-type carboxypeptidase activity | 8.01% | 3.05% | 4.95% | 1.95% | 2.33% | 4.89% | 6.21% | 7.40% | 7.10% | 2.71% | 4.86% |
| phosphatase activity | 3.78% | 5.03% | 1.65% | 4.94% | 3.80% | 2.76% | 3.91% | 4.63% | 1.83% | 3.49% | 3.58% |
| lipase activity | 5.80% | 2.36% | 1.65% | 0.90% | 2.76% | 5.60% | 0.23% | 1.34% | 1.22% | 1.13% | 2.30% |
| ammonium transmembrane transport | 5.48% | 1.72% | 1.65% | 0.00% | 3.36% | 4.09% | 0.00% | 0.09% | 0.20% | 5.76% | 2.24% |
| beta-galactosidase activity | 4.90% | 2.23% | 3.77% | 1.35% | 1.40% | 1.69% | 0.23% | 0.18% | 2.84% | 2.18% | 2.08% |
| glutathione transferase activity | 0.68% | 2.82% | 0.94% | 3.29% | 3.95% | 0.71% | 1.84% | 1.07% | 1.83% | 0.67% | 1.78% |
| water channel activity | 0.74% | 0.79% | 1.89% | 0.90% | 1.16% | 1.33% | 2.99% | 1.96% | 3.45% | 1.42% | 1.66% |
| chitinase activity | 4.98% | 2.29% | 2.36% | 1.20% | 2.01% | 3.47% | 0.00% | 0.00% | 0.00% | 0.00% | 1.63% |
| **Other** | **7.45%** | **5.73%** | **10.61%** | **5.39%** | **4.96%** | **9.61%** | **5.29%** | **3.65%** | **4.46%** | **5.76%** | 6.29% |
| *heat shock protein activity* | 1.69% | 0.84% | 2.12% | 0.90% | 1.57% | 1.42% | 2.30% | 1.25% | 0.00% | 1.75% | 1.38% |
| *fructose-bisphosphate aldolase activity* | 0.92% | 0.91% | 2.12% | 1.05% | 1.14% | 0.80% | 1.38% | 1.43% | 1.22% | 0.51% | 1.15% |
| *ribonuclease activity* | 0.83% | 1.18% | 0.94% | 0.75% | 0.96% | 2.22% | 1.61% | 0.62% | 1.42% | 0.98% | 1.15% |
| *phospholipase activity* | 1.53% | 0.43% | 1.65% | 0.90% | 0.00% | 0.98% | 0.00% | 0.00% | 0.00% | 0.36% | 0.58% |
| *actin filament* | 0.98% | 0.00% | 0.71% | 0.30% | 0.22% | 0.98% | 0.00% | 0.00% | 1.42% | 0.00% | 0.46% |
| *alternative oxidase activity* | 0.18% | 0.18% | 1.42% | 0.45% | 0.25% | 1.51% | 0.00% | 0.00% | 0.00% | 0.91% | 0.49% |
| *ATP:ADP antiporter activity* | 0.60% | 0.78% | 0.71% | 0.45% | 0.33% | 0.44% | 0.00% | 0.36% | 0.41% | 0.66% | 0.47% |
| *cinnamyl-alcohol dehydrogenase activity* | 0.71% | 1.41% | 0.47% | 0.60% | 0.48% | 0.53% | 0.00% | 0.00% | 0.00% | 0.59% | 0.48% |
| *thioglucosidase activity* | 0.00% | 0.00% | 0.47% | 0.00% | 0.00% | 0.71% | 0.00% | 0.00% | 0.00% | 0.00% | 0.12% |
