# Supplementary material for: Evaluating the adaptive evolutionary convergence of carnivorous plant taxa through functional genomics
Source: PeerJ. 2018 Jan 31;6:e4322. doi: 10.7717/peerj.4322 (PMC5797450; doi:10.7717/peerj.4322)
Supplement: Table S4 — Equivalent to main text Table 3, but using unadjusted data. “t” indicates the test statistic of an upper-tailed Student’s t-test. “p” indicates the p-value of this test. “ q” indicates a corrected p-value accounting for multiple comparisons, using Storey’s correction. Significance (“Sig.”) is indicated by bolding and with “*” for q < 0.05, “**” for q < 0.01, and “*** for q < 0.001. A non-bolded “.” indicates marginal values (q < 0.10), while “NS” indicates non-significance (q > 0.10). [file peerj-06-4322-s006.docx]

|  | **t** | **p** | **q** | **Sig.** |
| --- | --- | --- | --- | --- |
| Actin | -0.17 | 0.565 | 0.149 | *NS* |
| AltOx | **3.34** | **0.007** | **0.033** | ******* |
| AspPep | -0.29 | 0.609 | 0.152 | *NS* |
| ATP | 0.05 | 0.479 | 0.144 | *NS* |
| ATP_ADP | **2.91** | **0.013** | **0.033** | ******* |
| BGal | 1.94 | 0.054 | 0.068 | *.* |
| Chit | -2.27 | 0.972 | 0.194 | *NS* |
| CinAlc | 0.40 | 0.351 | 0.144 | *NS* |
| CystPep | 0.08 | 0.469 | 0.144 | *NS* |
| FrucBPA | 0.65 | 0.269 | 0.135 | *NS* |
| GlutTrans | 0.10 | 0.462 | 0.144 | *NS* |
| H2OChan | 1.42 | 0.098 | 0.082 | *.* |
| HeatShock | 0.14 | 0.448 | 0.144 | *NS* |
| Lipase | -0.12 | 0.546 | 0.149 | *NS* |
| LipTrans | 0.23 | 0.413 | 0.144 | *NS* |
| NHTrans | 0.67 | 0.270 | 0.135 | *NS* |
| Perox | -0.39 | 0.647 | 0.154 | *NS* |
| Phoslip | **2.57** | **0.022** | **0.037** | ******* |
| Phosp | 1.25 | 0.142 | 0.089 | *.* |
| Polygal | -0.67 | 0.739 | 0.161 | *NS* |
| ProtHomo | 1.47 | 0.091 | 0.082 | *.* |
| RiboNuc | -0.89 | 0.796 | 0.166 | *NS* |
| SerCarPep | -0.54 | 0.696 | 0.158 | *NS* |
| ThioGluc | 1.34 | 0.129 | 0.089 | *.* |
| Total | 0.03 | 0.489 | 0.144 | *NS* |
