# Supplementary material for: Evaluating the adaptive evolutionary convergence of carnivorous plant taxa through functional genomics
Source: PeerJ. 2018 Jan 31;6:e4322. doi: 10.7717/peerj.4322 (PMC5797450; doi:10.7717/peerj.4322)
Supplement: Table S5 — Equivalent to main text Table 4 but using unadjusted data. “t” indicates the test statistic of an upper-tailed Student’s t-test. “ p” indicates the p-value of this test. “ q” indicates a corrected p-value accounting for multiple comparisons, using Storey’s correction. Significance (“Sig.”) is indicated by bolding and with “*” for q < 0.05, “**” for q < 0.01, and “*** for q < 0.001. A non-bolded “.” indicates marginal values (q < 0.10), while “NS” indicates non-significance (q > 0.10). [file peerj-06-4322-s007.docx]

|  | *Genlisea aurea* | | | | *Drosera capensis* | | | | *Utricularia gibba* | | | | *Cephalotus follicularis* | | | |
| --- | --- | --- | --- | --- | --- | --- | --- | --- | --- | --- | --- | --- | --- | --- | --- | --- |
|  | **Z** | **p** | **q** | **Sig.** | **Z** | **p** | **q** | **Sig.** | **Z** | **p** | **q** | **Sig.** | **Z** | **p** | **q** | **Sig.** |
| Actin | 0.26 | 0.396 | 0.660 | *NS* | -0.66 | 0.746 | 0.554 | *NS* | -0.04 | 0.517 | 0.152 | *NS* | 0.13 | 0.448 | 0.189 | *NS* |
| AltOx | 0.85 | 0.198 | 0.476 | *NS* | **3.24** | **5.98E-04** | **9.56E-03** | ******** | 2.53 | **0.006** | **0.012** | ******* | **2.04** | **0.021** | **0.023** | ******* |
| AspPep | -0.71 | 0.760 | 0.792 | *NS* | 0.91 | 0.181 | 0.353 | *NS* | -0.34 | 0.635 | 0.164 | *NS* | -0.48 | 0.686 | 0.199 | *NS* |
| ATP | -2.02 | 0.978 | 0.797 | *NS* | 0.85 | 0.199 | 0.353 | *NS* | 1.31 | 0.094 | 0.079 | *.* | 0.05 | 0.482 | 0.189 | *NS* |
| ATP_ADP | 1.03 | 0.150 | 0.476 | *NS* | 2.80 | **2.55E-03** | **0.016** | ******* | 1.05 | 0.148 | 0.106 | *NS* | **3.06** | **0.001** | **0.002** | ******** |
| BGal | 1.97 | 0.024 | 0.163 | *NS* | 0.28 | 0.390 | 0.554 | *NS* | 0.62 | 0.269 | 0.142 | *NS* | **3.19** | **7.19E-04** | **2.15E-03** | ******** |
| Chit | -1.08 | 0.860 | 0.797 | *NS* | -1.10 | 0.865 | 0.554 | *NS* | -1.61 | 0.946 | 0.189 | *NS* | -0.47 | 0.682 | 0.199 | *NS* |
| CinAlc | 1.26 | 0.104 | 0.416 | *NS* | -0.39 | 0.653 | 0.554 | *NS* | 0.34 | 0.366 | 0.152 | *NS* | -0.32 | 0.625 | 0.198 | *NS* |
| CystPep | -2.69 | 0.996 | 0.797 | *NS* | 2.13 | 0.017 | 0.053 | *.* | 2.16 | 0.015 | 0.015 | *** | -1.17 | 0.880 | 0.235 | *NS* |
| FrucBPA | 1.56 | 0.059 | 0.295 | *NS* | -0.65 | 0.741 | 0.554 | *NS* | 0.75 | 0.227 | 0.142 | *NS* | -0.03 | 0.512 | 0.189 | *NS* |
| GlutTrans | 0.00 | 0.500 | 0.769 | *NS* | -0.31 | 0.623 | 0.554 | *NS* | -0.80 | 0.789 | 0.164 | *NS* | 1.36 | 0.087 | 0.083 | *.* |
| H2OChan | 0.83 | 0.203 | 0.476 | *NS* | 0.08 | 0.470 | 0.554 | *NS* | 0.57 | 0.284 | 0.142 | *NS* | 1.20 | 0.114 | 0.095 | *.* |
| HeatShock | 0.78 | 0.217 | 0.476 | *NS* | -0.81 | 0.792 | 0.554 | *NS* | 0.06 | 0.477 | 0.152 | *NS* | 0.26 | 0.398 | 0.189 | *NS* |
| Lipase | -0.14 | 0.556 | 0.792 | *NS* | -0.39 | 0.651 | 0.554 | *NS* | -0.23 | 0.591 | 0.164 | *NS* | 0.54 | 0.295 | 0.164 | *NS* |
| LipTrans | 0.71 | 0.238 | 0.476 | *NS* | -0.30 | 0.616 | 0.554 | *NS* | -0.55 | 0.709 | 0.164 | *NS* | 0.60 | 0.274 | 0.164 | *NS* |
| NHTrans | -0.81 | 0.792 | 0.792 | *NS* | 1.12 | 0.130 | 0.298 | *NS* | 3.18 | **7.40E-04** | **3.70E-03** | ******** | -0.75 | 0.773 | 0.215 | *NS* |
| Perox | -0.52 | 0.700 | 0.792 | *NS* | -0.43 | 0.667 | 0.554 | *NS* | 0.04 | 0.483 | 0.152 | *NS* | 0.21 | 0.416 | 0.189 | *NS* |
| Phoslip | 2.78 | 2.69E-03 | 0.054 | *.* | 0.09 | 0.464 | 0.554 | *NS* | 2.33 | **9.95E-03** | **0.012** | ******* | **2.32** | **1.03E-02** | **0.014** | ******* |
| Phosp | -1.96 | 0.975 | 0.797 | *NS* | 2.76 | 0.003 | 0.016 | *** | 2.40 | **0.008** | **0.012** | ******* | **3.01** | **1.29E-03** | **2.15E-03** | ******** |
| Polygal | -0.64 | 0.740 | 0.792 | *NS* | -1.07 | 0.859 | 0.554 | *NS* | 0.42 | 0.337 | 0.152 | *NS* | -0.10 | 0.539 | 0.189 | *NS* |
| ProtHomo | 2.03 | 0.021 | 0.163 | *NS* | 0.28 | 0.390 | 0.554 | *NS* | 0.22 | 0.413 | 0.152 | *NS* | 0.92 | 0.179 | 0.132 | *NS* |
| RiboNuc | -0.76 | 0.777 | 0.792 | *NS* | -0.14 | 0.557 | 0.554 | *NS* | -0.46 | 0.678 | 0.164 | *NS* | -0.17 | 0.568 | 0.189 | *NS* |
| SerCarPep | -0.28 | 0.609 | 0.792 | *NS* | -0.98 | 0.838 | 0.554 | *NS* | -0.80 | 0.790 | 0.164 | *NS* | 0.83 | 0.203 | 0.136 | *NS* |
| ThioGluc | 0.40 | 0.345 | 0.627 | *NS* | 2.38 | 8.63E-03 | 0.035 | *** | -0.74 | 0.771 | 0.164 | *NS* | **4.63** | **1.82E-06** | **1.21E-05** | ********** |
| Total | -1.22 | 0.889 | 0.797 | *NS* | 1.25 | 0.106 | 0.283 | *NS* | 0.02 | 0.490 | 0.152 | *NS* | 0.02 | 0.491 | 0.189 | *NS* |
